# Spinning-disc confocal microscopy in the second near-infrared window (NIR-II)

**DOI:** 10.1038/s41598-018-31928-y

**Published:** 2018-09-13

**Authors:** Vitalijs Zubkovs, Alessandra Antonucci, Nils Schuergers, Benjamin Lambert, Andrea Latini, Raino Ceccarelli, Andrea Santinelli, Andrii Rogov, Daniel Ciepielewski, Ardemis A. Boghossian

**Affiliations:** 1 Institute of Chemical Sciences and Engineering (ISIC), École Polytechnique Fédérale de Lausanne (EPFL), Lausanne, CH-1015 Switzerland; 2 CrestOptics S.p.A, Rome, 00167 Italy; 3 Nikon GmbH, Swiss Branch, Egg, 8132 Switzerland

## Abstract

Fluorescence microscopy in the second near-infrared optical window (NIR-II, 1000–1350 nm) has become a technique of choice for non-invasive *in vivo* imaging. The deep penetration of NIR light in living tissue, as well as negligible tissue autofluorescence within this optical range, offers increased resolution and contrast with even greater penetration depths. Here, we present a custom-built spinning-disc confocal laser microscope (SDCLM) that is specific to imaging in the NIR-II. The SDCLM achieves a lateral resolution of 0.5 ± 0.1 µm and an axial resolution of 0.6 ± 0.1 µm, showing a ~17% and ~45% enhancement in lateral and axial resolution, respectively, compared to the corresponding wide-field configuration. We furthermore showcase several applications that demonstrate the use of the SDCLM for *in situ*, spatiotemporal tracking of NIR particles and bioanalytes within both synthetic and biological systems.

## Introduction

A variety of near-infrared (NIR) fluorophores, such as single-walled carbon nanotubes (SWCNTs), quantum dots (QDs), inorganic nanoparticles, fluorescent proteins, and dyes, have been specifically developed for imaging and optical sensing applications^1–7^. These fluorophores span wavelengths throughout the NIR window from 700 to 1350 nm^8–10^. Since biological pigments like lipopigments and porphyrins are autofluorescent between 650 and 950 nm^5,11,12^, and water strongly absorbs light between 1350 and 1500 nm^8,13^, fluorophores that lie within the second NIR range (NIR-II) from 1000 to 1350 nm specifically benefit from low cross-contamination and increased tissue penetration, permitting deep-tissue imaging and sensing applications (Table 1). NIR organic dyes in particular also benefit from biocompatibility and relatively simple conjugation methods that can be used on a variety of different substrates, including proteins and polymers. However, photobleaching of the dyes limits long-term monitoring; for example, the fluorescence intensity of IR-1061, a commercially available NIR dye, has been shown to drop to 50% of its initial value after 1250 s of continuous illumination at 808 nm when dissolved in dimethyl sulfoxide^14^. Although synthetic nanoparticles such as QDs are also susceptible to photobleaching in addition to cytotoxicity that limits their use *in vivo*, they enjoy advantages such as greater emission tunability^5^. Alternatively, certain synthetic nanoparticles such as SWCNTs show indefinite photostability and can be functionalized to achieve improved biocompatibility. Despite quantum yields on the order of just 0.1%, the fluorescence tunability, indefinite stability, and improved biocompatibility make SWCNTs a formidable option for *in vivo* imaging and sensing applications^15,16^.

**Table 1:** Fluorophores with emission maxima in the NIR-II window between 1000 and 1350 nm. The fluorophores are listed by excitation wavelength maxima in ascending order.

Fluorophore	Chemical Structure	Ex. (nm)	Em. (nm)	QY (%)	Year	Ref.
(7,6)-SWCNT		650	1120	0.1	1991	^16^
CH-4T		740	1000	11	2017	^54^
CH1055		750	1055	0.3	2015	^2^
H1		800	1100	2	2017	^55^
Q4		880	1100	—	2016	^56^
CQS1000		885	1000	—	2016	^56, 57^
IR-1061		1074	1132	1.7	2014	^14^
IR-26		1080	1190	0.5	1981	^54, 58^
PbS QDs (Ø 4.4 nm)		1250	1280	45	1994	^59^
PbSe QDs (Ø 4.3 nm)		1300	1350	41	2001	^59– 61^

Several existing setups are capable of imaging the expanding number of fluorophores in the NIR-II window. Similar in construction to conventional visible range optical microscopes, these setups are mostly distinguished by the use of a NIR-specific camera with an InGaAs-coated sensor. Recent advancements in wide-field deconvolution, laser-scanning confocal and super-resolution microscopy offer promising approaches to achieving high-resolution images of NIR-II fluorophores^17–20^. However, these methods require relatively long acquisition times that limit their use for real-time monitoring^19^. An alternative approach is spinning-disc confocal light microscopy (SDCLM), which is a high-speed optical sectioning technique widely used in biological sciences^21^. Whereas existing commercially available setups are largely limited to confocal fluorescence imaging in the visible region of the optical spectrum, this manuscript presents a spinning-disc confocal setup tailored for imaging in the NIR-II window. The resolution limits and acquisition speed of the microscope are determined and subsequently used to image SWCNTs. The advantages of NIR SDCLM imaging is exemplarily demonstrated in three distinct applications: single-particle tracking of NIR fluorescent nanoparticles in solution, spatial distribution of internalized nanoparticles within an organelle, and optical detection of glucose using immobilized SWCNT-based sensors.

## Results and Discussion

### Spatial resolutions of NIR microscopes

**Figure 1:**
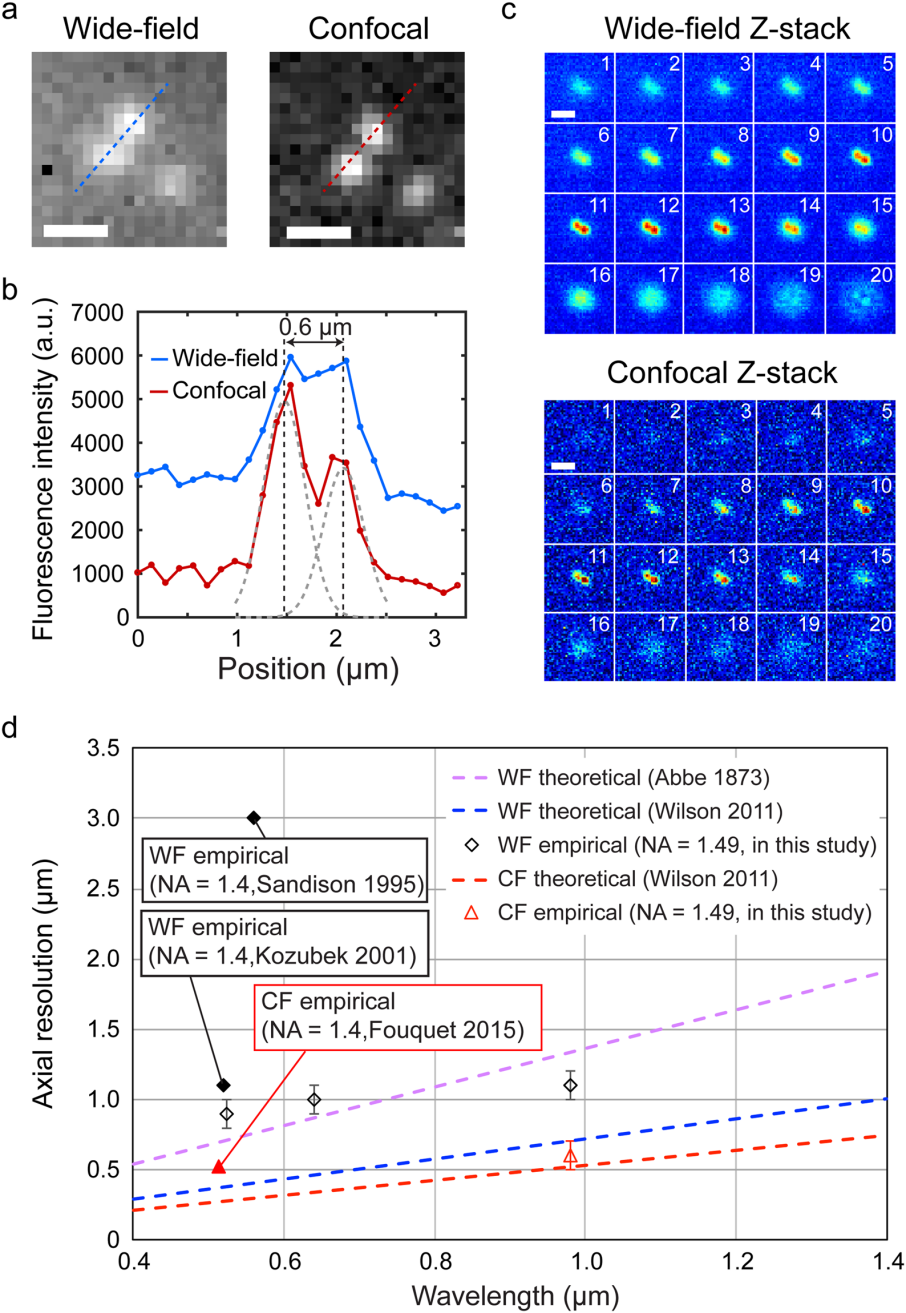
Comparison of wide-field (WF) and spinning-disc confocal (CF) NIR microscope images. (**a**) Wide-field (left) and confocal (right) images of 186 ± 48 nm NIR fluorescent beads. Scale bar = 1 µm. (**b**) Intensity profiles of the cross-sections indicated in (**a**) by dashed lines. (**c**) Z-stack projection of wide-field (top) and confocal (bottom) images of the fluorescent beads. Scale bar = 2 µm. (**d**) Wavelength dependence of theoretical and determined axial resolutions. The theoretical resolutions were calculated according to Equation 4 (purple dashed line) and Equation 5 for wide-field F_z_ = 0.89 (blue dashed line) and confocal F_z_ = 0.66 (red dashed line) cases, where NA = 1.49, n = 1.515^25,26,51^. Empirical resolutions in the wide-field settings (white diamonds) were determined using enhanced yellow fluorescent protein (excitation at 480 nm, emission at 525 nm), quantum dots (excitation at 425 nm, emission at 640 nm), and NIR beads (excitation at 780 nm, emission at 980 nm), and they were compared to empirical data in the literature (shaded diamonds)^23,52^. The empirical resolution in the confocal settings (red triangle) was determined using NIR beads (excitation at 780 nm, emission at 980 nm), and it was compared to empirical data in the literature (shaded triangle)^53^.

Our SDCLM setup consists of a spinning-disc module coupled to a cooled InGaAs camera installed on a Nikon Eclipse Ti-E microscope body (see Supplementary Information, Figure S1). Lenses in the spinning-disc confocal unit were coated with a NIR anti-reflective layer to maximize photon throughput (see Supplementary Information, Figure S2). The disc can be removed from the optical path to image a specimen in the wide-field configuration. We compared images of NIR fluorescent beads (186 ± 48 nm diameter) recorded in the wide-field and confocal microscope configurations (Fig. 1). The beads were immersed in oil with a refractive index of n = 1.515. As shown in Fig. 1a, the confocal image (right) offers better image contrast and clearer distinction between two neighboring beads in comparison to the wide-field image (left). The intensity profiles of the lateral cross-sections in Fig. 1b show that the two beads remain unresolved in the wide-field image when the distance between them is 0.6 μm, while they are clearly separated by confocal imaging. The full-width at half-maximum (FWHM) of lateral cross-sections of single beads are 0.6 ± 0.1 µm and 0.5 ± 0.1 µm in wide-field and confocal modes, respectively (see Supplementary Information, Table S1). According to the Abbe equation, the theoretical lateral resolution limit in the wide-field configuration is r_xWF_ = 0.33 µm (Equation 1, where the wavelength is λ_EM_ = 980 nm and the numerical aperture is NA = 1.49)^22^.

1 $${r}_{xWF}=0.5\frac{{\lambda }_{EM}}{NA}$$

We note that this theoretical resolution does not precisely account for all types of aberrations present in an optical setup nor signal-to-noise restrictions, and it is therefore typically lower than the experimental values^23,24^. The lateral resolution is improved within the confocal microscope by limiting the out-of-focus emissions using small confocal pinholes with a diameter of 0.5 Airy units (AU) (or 60 µm), with one AU at the disc plane equal to 120 µm (Equation 2, λ_EM_ = 980 nm, NA = 1.49, magnification = 150×). Hence, the theoretical lateral resolution of the confocal microscope is calculated to be r_xCF_ = 0.26 µm (Equation 3, the lateral FWHM pre-factor is F_x_ = 0.40)^25^.

2 $${d}_{x}=AU=1.22\frac{{\lambda }_{EM}}{NA}$$

3 $${r}_{x}={F}_{x}\frac{{\lambda }_{EM}}{NA}$$

The empirical lateral resolution is approximately half that of the theoretical limit. This difference can be explained by spherical aberrations of the optical lenses and a mismatch in the refractive indices of the sample media and coverglass^23,26^.

We determined the axial resolution of the imaging system through longitudinal scanning of the fluorescent beads (Fig. 1c) measuring the FWHM of emission intensities in the Z-direction. The axial resolution of 1.1 ± 0.1 µm in the wide-field configuration is surpassed by a resolution of 0.6 ± 0.1 μm in the confocal configuration (see Supplementary Information, Table S2). These values are close to the lowest experimental values of ~1.0 µm and ~0.5 µm reported for wide-field and confocal fluorescence microscopes in the visible light region^27^. The theoretical axial resolution for the confocal configuration is r_zCF_ = 0.52 µm (Equation 5, with F_z_ equal to 0.66 for the 60 µm spinning-disc pinholes), which is slightly smaller than the experimental value (Fig. 1d). The improvement in axial resolution by ~45% in the confocal mode therefore offers enhanced precision for imaging sub-micrometer objects, as reported later in this article.

4 $${r}_{zWF}=\frac{2\cdot {\lambda }_{EM}\cdot n}{N{A}^{2}}$$

5 $${r}_{z}={F}_{z}\frac{{\lambda }_{EM}}{n-\sqrt{{n}^{2}-N{A}^{2}}}$$

### Image acquisition speed in NIR microscopy

In comparison to point-scanning NIR confocal microscopes or NIR super-resolution systems, SDCLMs allow faster image acquisition. Capturing an area of 50 × 50 µm with a NIR uPAINT setup takes more than two seconds^28^. The confocal microscope allows for high spatial resolution while the spinning-disc maintains fast imaging acquisition speeds. The disc rotates at 15000 RPM (250 Hz), and it is designed to achieve the maximal theoretical acquisition speed of ~18000 fps, which is equivalent to a minimal exposure time of ~50 μs. However, the NIRvana 640 InGaAs camera in this setup limits the acquisition time to 110 fps or 9 ms. When imaging NIR beads, minimum capturing times of 20 fps or 50 ms (see Supplementary Information, Figure S3) were selected to achieve a desirable signal-to-noise ratio of more than 40^29^. Accordingly, the predominant factor limiting acquisition speed in this SDCLM setup is the brightness of the NIR fluorophores.

### Temporal imaging of diffusing NIR beads

**Figure 2:**
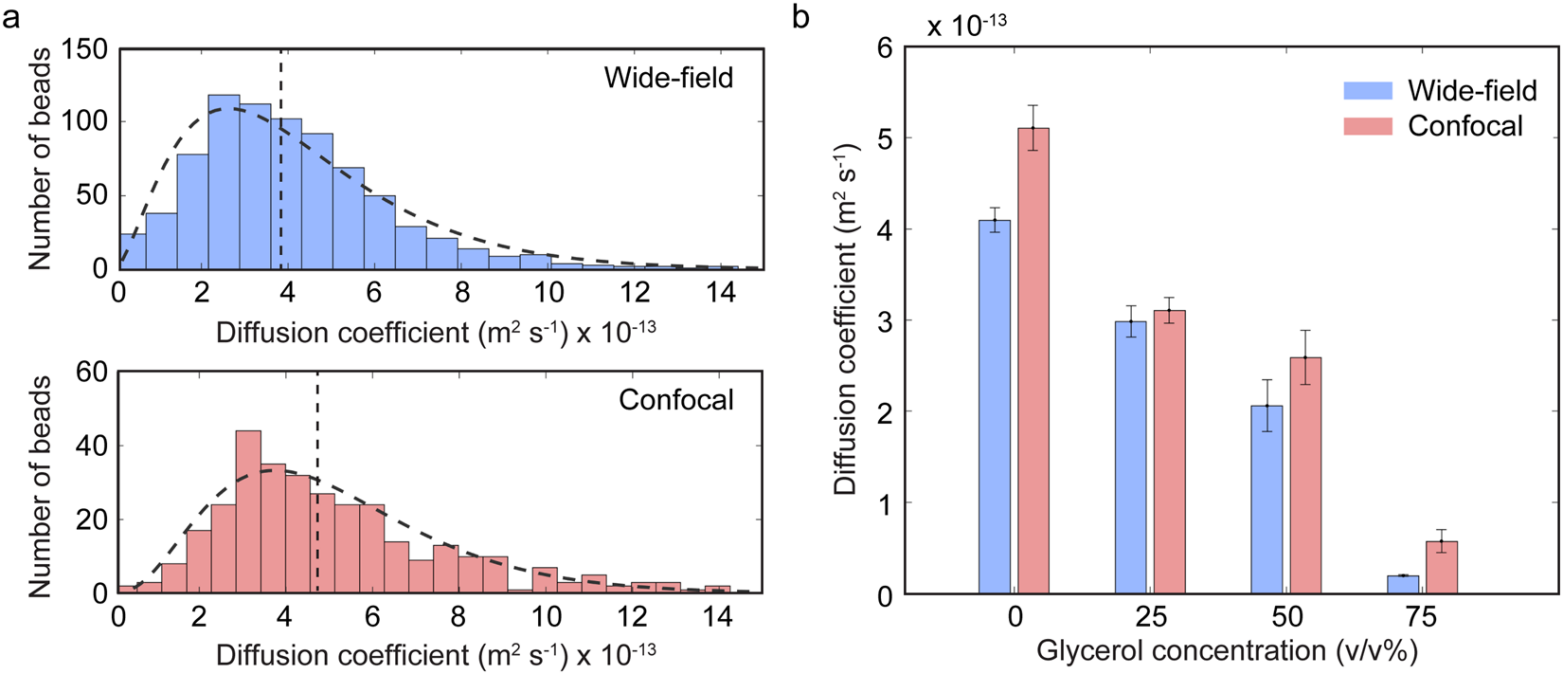
Tracking of single NIR beads in water and viscous glycerol solutions. (**a**) Distribution of calculated diffusion coefficients of NIR beads in water. The dashed lines are fits to the beta distribution function. Median values for the wide-field and confocal distributions are 3.72 × 10^−13^ m^2^/s and 4.67 × 10^−13^ m^2^/s (vertical dashed lines), respectively. (**b**) Mean diffusion coefficients of NIR beads in 0, 25, 50, 75 v/v% aqueous glycerol solutions (error bars represent 95% confidence intervals). Between 300 and 800 bead trajectories were analyzed for each set of conditions.

We tracked the 2D Brownian motion of NIR fluorescent beads in viscous solutions containing different concentrations of glycerol using both wide-field and confocal configurations (Fig. 2). The particle trajectories were used to determine diffusion coefficients (D) according to the formula^30^,

6 $${\rm{MSD}}=4{\rm{Dt}}$$

where MSD is the mean-squared displacement and *t* is time. The median diffusivity in water is ~25% higher based on trajectories from the confocal configuration compared to those obtained from the wide-field configuration (Fig. 2a). This difference is attributed to the more precise tracking of bead movement in the axial direction and thinner plane of the confocal configuration. To minimize the contribution of particles traversing the axial direction, which leads to underestimation of particle diffusivity, we only consider tracks that are longer than 0.6 s (12 frames) in calculating the average and median diffusivities. As a consequence, the wide-field trajectories slightly underestimate the diffusivity since particles move in the axial direction for longer distances (and time) before leaving the plane. The mean diffusion coefficient was calculated for solutions containing different concentrations of glycerol (Fig. 2b). According to the Stokes-Einstein equation^31,32^,

7 $$D=\frac{{k}_{B}\cdot T}{6\cdot \pi \cdot \eta \cdot r}$$

where k_B_ is Boltzmann’s constant, T is temperature, η is dynamic viscosity, and r is the radius of the spherical particle, the diffusivity is expected to vary inversely with solution viscosity. At 20 °C, the corresponding diffusivities for the glycerol solutions at 0, 25, 50, and 75 v/v%, with dynamic viscosities between 0.001 kg/(m·s) for pure water and 0.055 kg/(m·s) for 75 v/v% glycerol, are predicted to range between 18.3–30.9 × 10^−13^, 7.3–12.4 × 10^−13^, 2.1–3.5 × 10^−13^, and 0.3–0.6 × 10^−13^ m^2^/s, respectively. Though these values largely lie within the same order of magnitude as the experimental values calculated from the MSD trajectories, the experimental values tend to lie below predicted values, particularly for lower glycerol concentrations where particles are expected to diffuse much more quickly. This observation suggests that limited frame rate may result in the underestimation of diffusivities for faster diffusing particles. Nonetheless, the values reported herein for the confocal measurements are closer to the theoretical value than the wide-field measurements, which further underestimate the measured diffusivities.

### 3D localization of SWCNTs in plant chloroplasts

**Figure 3:**
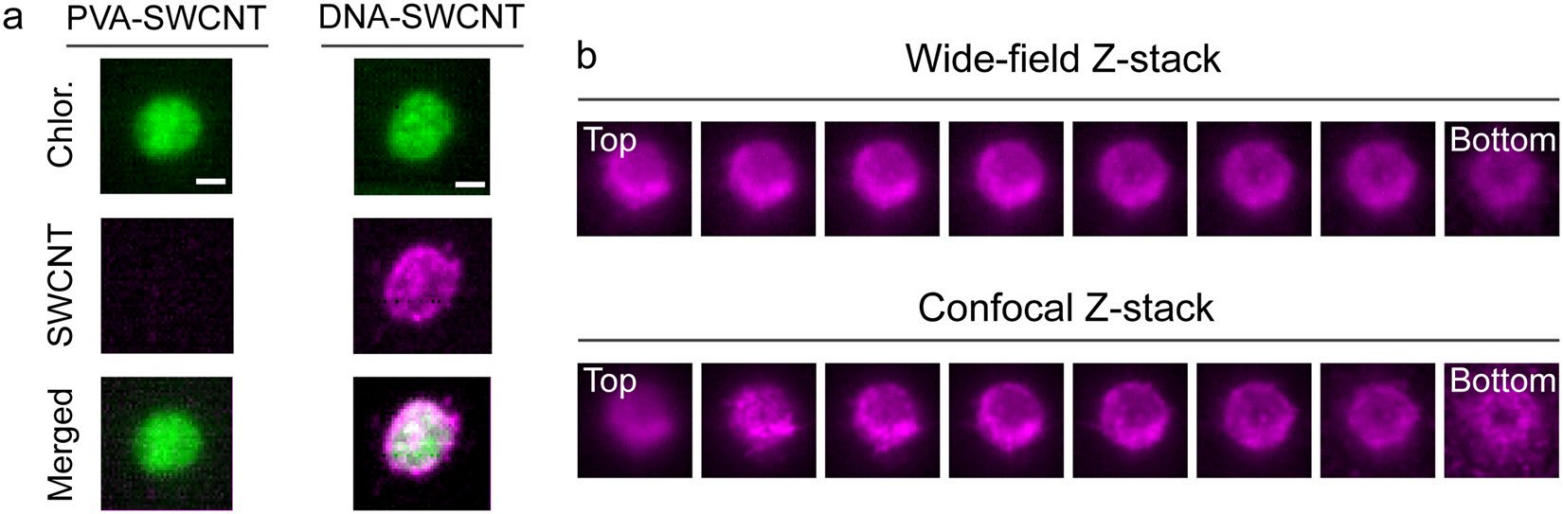
Internalization of functionalized SWCNTs in a chloroplast. (**a**) Autofluorescence (green, excitation at 640 nm, emission above 950 nm), SWCNT fluorescence (violet, excitation at 780 nm, emission above 980 nm), and merged confocal images recorded after incubation with PVA-SWCNTs and DNA-SWCNTs. Scale bar = 2 µm. (**b**) NIR wide-field and confocal Z-stack images of DNA-SWCNTs within an isolated chloroplast (step size = 0.4 µm, excitation at 780 nm, emission above 980 nm).

The improved SDCLM resolution offers a promising basis for enhanced *in vitro* and *in vivo* imaging. We explored this prospect by imaging functionalized SWCNTs within photosynthetic chloroplasts^33^. Chloroplasts are organelles that autofluoresce in the visible range of the spectrum, benefitting in particular from imaging techniques that use NIR dyes with distinct fluorescent wavelengths. Although fluorescent properties of SWCNTs are attractive for NIR imaging inside living cells and organelles, their uptake can strongly depend on surface functionalization and the properties of wrapping polymers. Previous studies have shown that wrappings conferring a strongly positive or negative zeta potential allow SWCNTs to traverse the outer membrane of chloroplasts^34^. In agreement with these findings, our measurements show that extracted chloroplasts do not internalize polyvinyl alcohol-wrapped SWCNTs (PVA-SWCNTs), which are predicted to have an almost neutral surface zeta potential^35^, while DNA-wrapped SWCNTs (DNA-SWCNTs) that exhibit a large negative zeta potential (approximately −45 mV in PBS pH 7) readily localize within the organelles (Fig. 3a). The NIR imaging shows an inhomogeneous localization of SWCNTs within the chloroplast, with the sharper resolution offered in the confocal z-stack images revealing a rather granular distribution of SWCNT fluorescence, particularly in areas with low SWCNT fluorescence (Fig. 3b). The axial distribution of DNA-SWCNTs in chloroplasts could not be readily discerned in the confocal Raman images reported in previous studies because of a limited axial resolution of 2 µm^34^.

### Spatiotemporal glucose detection in an agarose gel

**Figure 4:**
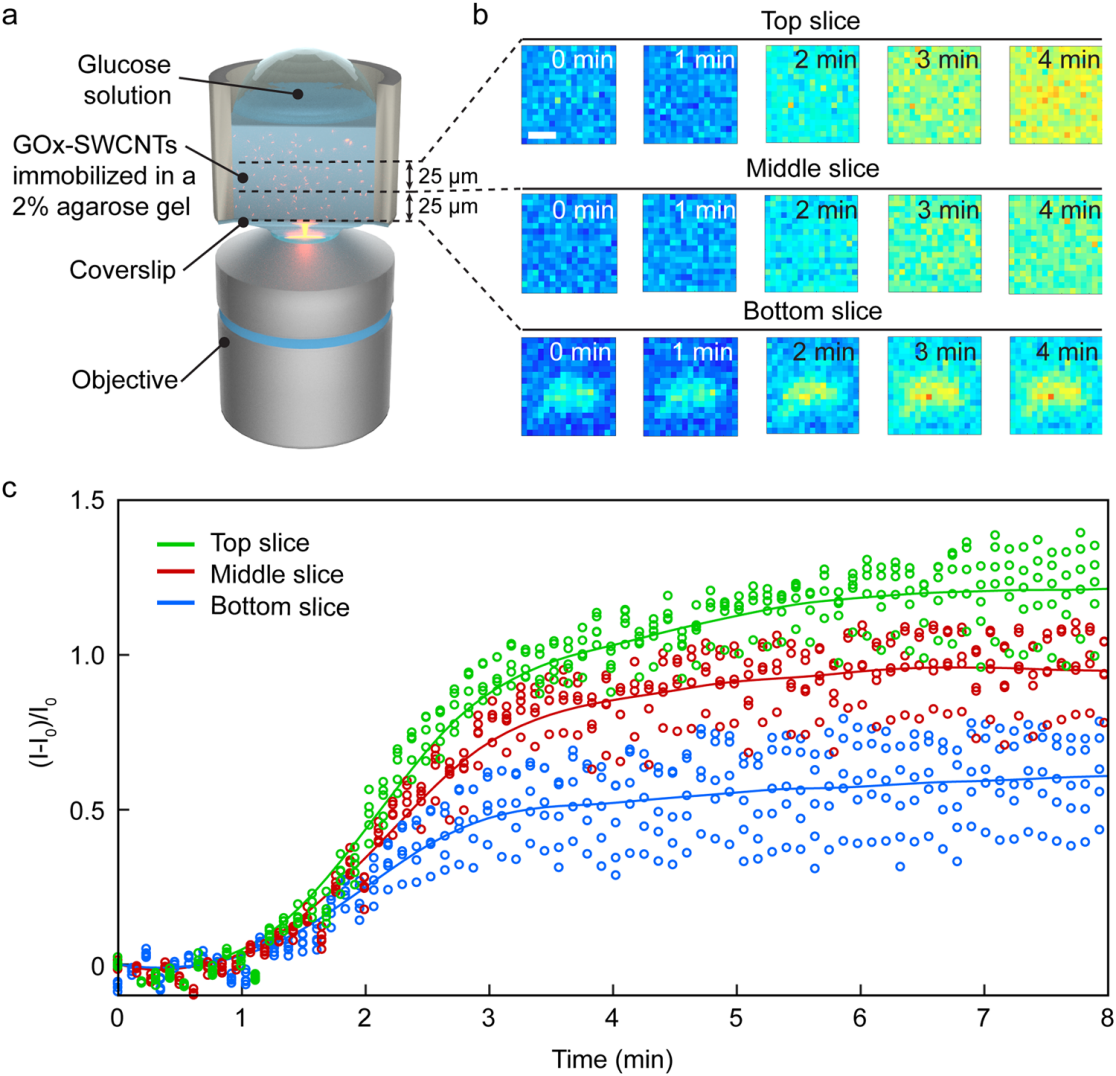
Spatiotemporal response of immobilized GOx-SWCNT sensors to glucose. (**a**) Schematic of the setup. GOx-SWCNTs sensors were embedded in a 2% agarose gel within a glass-bottom well. (**b**) Confocal planar images (step size = 25 µm) of representative clusters with NIR fluorescent GOx-SWCNTs (excitation at 660 nm, emission above 980 nm). The observed fluorescence increase of the GOx-SWCNT sensors over time was triggered by adding enough glucose solution to yield a final concentration of 15 mM. Scale bar = 2.5 µm. (**c**) Normalized ((I-I_0_)/I_0_) fluorescence intensity change of five GOx-SWCNT clusters from each axial position after addition of glucose (empty circles), where I is intensity and I_0_ is initial intensity. The lines show average intensity changes at the top (green), middle (red), and bottom (blue) slices after applying locally weighted scatterplot smoothing (LOWESS).

SWCNTs are typically functionalized with biomolecules such as DNA, RNA, and proteins to enhance their fluorescence response towards specific analytes^36–39^. One example is the use of glucose oxidase-wrapped SWCNTs (GOx-SWCNTs), which have been previously shown to undergo a specific increase in fluorescence intensity in response to glucose^40^. Such biosensors are often encapsulated in gel matrices such as agarose prior to implantation for *in vivo* monitoring. Ensemble measurements of the implanted SWCNT sensors are measured despite gel diffusion limitations that may contribute to a heterogeneous SWCNT response. Minimizing this heterogeneity through facilitated diffusion can therefore improve sensor response. The spatiotemporal distribution of SWCNTs has been studied previously, for example, by Galassi *et al*., who imaged SWCNTs distributed within an agarose gel^41^. However, monitoring of the sensor response in 3D, particularly in the axial direction, has not yet been reported. We therefore performed *in situ* glucose monitoring within a 120 µm × 100 µm × 50 µm gel sample containing GOx-SWCNT sensors in a 2 wt% agarose gel (Fig. 4a). The GOx-SWCNT sensors were continuously excited with a 660 nm laser, and the fluorescence intensity of the sensors was monitored over multiple confocal planes to track the permeation of glucose in the axial direction. The fluorescence intensity of GOx-SWCNTs in all confocal planes ultimately increases in response to the addition of 15 mM glucose (Fig. 4b,c). Provided that the size of a glucose molecule (3.6 Å^42^) falls well below the average pore size of the agarose gel (~120 nm for 2% agarose gel), the matrix is expected to allow the glucose molecules to freely diffuse in the gel^43^. Assuming a diffusion coefficient of 5.73 × 10^−10^ m^2^/s^44^, the glucose diffusion time through a 50 µm layer of an agarose gel is ~2.2 s, which is expected to occur within one complete scan in the axial direction (the integration time for a single frame is 0.5 s; the Z-stack acquisition time is 4.6 s). The continuous diffusion of glucose from the surface into the gel creates a concentration gradient along the axial direction, as shown in Fig. 4c, which illustrates the NIR fluorescence increase at the top, middle slice, and bottom slices, consecutively. Although axial tracking of glucose permeation can be achieved through larger step sizes in the wide-field configuration, the spinning-disc confocal NIR microscope reduces the thickness of the sectioning by factor of ~2, allowing more precisely controlled and localized SWCNT imaging in the axial direction.

## Conclusions

We developed a spinning-disc confocal setup designed for imaging in the NIR-II window. The setup shows a ~17% and ~45% enhancement in lateral and axial resolutions, respectively, achieving a lateral resolution of 0.5 ± 0.1 µm and an axial resolution of 0.6 ± 0.1 µm. The enhancement in the resolution allows a more precise visualization of NIR nanoparticles within biological structures as small as plastids or bacterial cells. Confocal images of DNA-SWCNTs internalized in a single chloroplast reveal an inhomogeneous, granular distribution of nanoparticles in the organelle. This setup was also used to track the Brownian motion of NIR fluorescent beads in 0, 25, 50, and 75 v/v% aqueous glycerol solutions in wide-field and confocal modes. The diffusivities calculated from both modes fall within the range of expected theoretical diffusivities, with the diffusivities calculated from the wide-field measurements further underestimating the diffusion coefficient compared to those calculated from the confocal measurements. This discrepancy may arise from the thicker imaging plane of the wide-field setup, which accounts more for movement in the axial direction. Finally, the setup was also used to image the axial permeability of an analyte, in this case glucose, by monitoring the NIR fluorescence response of immobilized sensors within a gel matrix.

To further improve the spatial resolution and overcome the Abbe diffraction limit, the confocal system can be combined with Image Scanning Microscopy (ISM). Azuma and Kei demonstrated that the conventional spinning-disc confocal microscope can be combined with ISM to improve the lateral resolution over the wide-field by 27%^45^. In this work, the setup achieved a 17% increase in lateral resolution in the NIR light region, which could be enhanced using ISM as well as deconvolution of the point spread function (PSF). The NIR confocal imaging of photostable SWCNTs is suited for the extended acquisition times required for ISM (typically 1 to 25 s per frame) and would profit from the NIR transparency of biological tissues^46^. Future improvements to the setup also include placement of a piezoelectric axial positioning stage instead of the motorized stage, which is expected to increase the z-sectioning speed and resolution. Also, NIR sensors with greater QY will reduce the image acquisition time and take advantage of the higher frame rate achievable by the NIRvana camera. These improvements are particularly beneficial for applications that require local analyte detection within or on a cell, such as the detection of local dopamine release in axons.

Nonetheless, the three demonstrations highlighted with the current configuration illustrate the use of the setup for applications that not only require increased resolution of NIR particle distribution, but also NIR tracking of moving nanoparticles and axial tracking of analytes within immobilized 3D matrices. Though these three demonstrations provide only a glimpse of the possible applications unlocked with a NIR SDCLM, they lend a convincing basis for expanded studies on spatiotemporal monitoring for *in vitro and in vivo* imaging and sensing that include, for example, transient nanoparticle uptake and localization measurements in living cells and biological tissue.

## Materials and Methods

### Wide-field and confocal NIR fluorescence microscope

The 640, 660, and 780 nm continuous wave (CW) laser light sources (Triline Laser Bank, Cairn Research) are coupled to a Nikon Eclipse Ti-E microscope body by an optical fiber (FT1500 EMT, 0.39 NA) (see Supplementary Information, Figure S1). Both 780 ± 5 nm band-pass and 830 nm short-pass filters (Semrock) were installed in front of the fiber output. The laser light passes through a spinning-disc confocal unit (X-light, CrestOptics) that includes discs of spinning arrays with pinholes (∅ 60 µm) and lenses coated with a NIR anti-reflection layer (transmittance >60% in the wavelength range from 0.7 to 1.2 µm, see Supplementary Information, Figure S2). The excitation and emission beams are split by a dichroic mirror. When the microscope was operated in the wide-field configuration, the confocal assembly was removed from the light path. Samples mounted in the XYZ-translational stage were all illuminated through a TIRF Apo 100 × 1.49 NA oil immersion objective (Nikon Instruments) and 1.5x tube-lens. The fluorescence signal is collected in the epi-direction through a 980 ± 15 nm band-pass (Chroma Technology) or a 980 nm long-pass (BLP01-980R-25, Semrock) filter by a cooled indium gallium arsenide (InGaAs) camera (NIRvana 640 ST, Princeton Instruments). The laser power at the sample plane in the wide-field and confocal configurations are 2.6 W·cm^−2^ and 1.8 W·cm^−2^, respectively. Images were acquired using the Nikon NIS-Elements software (Nikon Instruments).

### Imaging of NIR fluorescent beads

Fluorophorex™ NIR fluorescent polystyrene beads with a mean diameter of 186 ± 48 nm were purchased from Phosphorex Inc. Beads were diluted to ~3 × 10^10^ particles per mL and 5 µL of the sample were spin-coated on a glass coverslip at 3000 rpm for 30 s following sonication (Polos SPIN150i, Semiconductor Production Systems). A drop of immersion oil (refractive index 1.515 at 23 °C, Type A, Nikon) was added on top of the coated layer, and bead fluorescence was imaged using an axial step size of 0.1 µm with 780 nm laser excitation and a 980 ± 15 nm band-pass filter. The optical resolution of the setup was determined from the lateral and axial FWHM fits of the imaged beads using the PSFj software^47^.

### Wide-field imaging in the visible light region

Glass coverslips were spin-coated with either 5 µL of enhanced yellow fluorescence proteins (eYFPs) (expressed and purified from genetically modified *Escherichia coli* cells) in a buffered solution or 5 µL of a quantum dot suspension (QD, Qdot 655 ITK amino(PEG), Life technologies corporation). Sample fluorescence was imaged in the wide-field configuration using an EMCCD camera (the objective with NA = 1.49 and iXon Ultra Andor camera) using 480 ± 15 nm excitation (Optoscan, Cairn) with a 525 ± 25 nm band-pass filter (Semrock) for eYFP or 425 ± 15 nm excitation with a 640 ± 15 nm band-pass filter (Semrock) for the QDs. The optical resolutions of the setup were determined from the lateral and axial FWHM fits of the imaged beads using PSFj software.

### Diffusion coefficients of the NIR fluorescent beads in different concentrations of glycerol

NIR beads were sonicated and suspended in water and in 25, 50, and 75 v/v% glycerol solutions (99.5% glycerol, Carl Roth) to a concentration of ~3 × 10^10^ particles per mL. 50 µL of each suspension were deposited in a glass-bottom petri dish (35 mm, ibidi). The fluorescence emission of the diffusing NIR beads was recorded over 5 min through an Apo 40 × 1.3 NA objective (Nikon Instruments) and a 1x tube lens with a frame rate of 20 fps. All experiments were performed at 20 °C. The particle displacement tracks and diffusion coefficients were determined from 2D image stacks using the ParticleTracker plugin of Fiji^48^. In the calculations of the average and median distributions of the diffusion coefficients, we considered only particles with diffusion trajectories longer than 12 frames (or 0.6 s), and the total number of frames per track was limited to 1000.

### Uptake of SWCNTs by isolated plant chloroplasts

Chloroplasts were isolated from commercially available spinach leaves as described previously^33^. 15 g of spinach leaves were ground using a mortar and pestle in 30 mL of ice-cooled chloroplast isolation buffer (0.05 M sodium-phosphate buffer (pH 7.3) with 0.4 M sucrose and 0.01 M KCl). Next, the suspension was centrifuged at 200 × g for 1 min at 4 °C to pellet unbroken cells and fragments. The supernatant was collected and transferred to a 50 mL tube. Centrifugation of the solution was repeated three times at 1500 × g for 10 min at 4 °C, and pellets with chloroplasts were stored in the dark at 4 °C. Freshly isolated chloroplasts at a concentration of 5 mg chlorophyll·L^−1^ were incubated for 15 minutes with 10 mg·L^−1^ suspensions of DNA- or PVA-SWCNTs and imaged by the NIR confocal microscope. The DNA- and PVA-SWCNT suspensions were prepared according to previously reported protocols^49,50^.

### Glucose detection in an agarose gel with GOx-SWCNT sensors

50 mg of SWCNTs (single-walled carbon nanotube – (7,6) chirality, 704121, Sigma Aldrich) were suspended in 50 mL of 2 wt% sodium cholate (Sigma Aldrich) in phosphate buffered saline (PBS pH 7.4, gibco®, Life Technologies). The suspension was sonicated for 30 min at 1% amplitude (1/4″ tip, Q700 Sonicator, Qsonica) in an ice bath and centrifuged at 164,000 × g for 4 h (Optima XPN-80 Ultracentrifuge, Beckman Coulter) to remove aggregates. Next, 30 mg of GOx from *Aspergillus niger* (Type II, Sigma Aldrich) was added to 1 mL of the sodium cholate-suspended SWCNTs and dialyzed in a 14 kDa cutoff dialysis tube (cellulose membrane, D9777, Sigma Aldrich) against 1.5 L of PBS at 4 °C. The GOx-SWCNT suspension was subsequently transferred to a 300 kDa dialysis device (Spectra/Por® Float-A-Lyzer®, Spectrum Laboratories) and dialyzed overnight at 4 °C. 10 µL of the GOx-SWCNTs was mixed with 10 µL of a warm (40–50 °C) 4% agarose gel in PBS (ultrapure Agarose, invitrogen), and 10 µL of this mixture were drop-casted in a well with a glass-bottom device fabricated from polydimethylsiloxane (PDMS). 50 µL of PBS was added on the top of the gel to prevent drying. The sample was mounted on the microscope’s specimen holder, and 60 µL of 30 mM glucose (ß-D-glucose, AB 136302, abcr) in PBS was added to the well. The fluorescence signal was recorded in the confocal microscope with an acquisition time of 0.5 s and scan rate of 2.3 s between Z-slices (excitation at 660 nm, long-pass 980 nm emission filter). The time-lapse Z-stacks were further analyzed using a customized MATLAB script.

## Electronic supplementary material

Supplementary Information
