# Supplementary material for: Spinning-disc confocal microscopy in the second near-infrared window (NIR-II)
Source: Sci Rep. 2018 Sep 13;8:13770. doi: 10.1038/s41598-018-31928-y (PMC6137042; doi:10.1038/s41598-018-31928-y)
Supplement: Supplementary file 1 — Supplementary Information [file 41598_2018_31928_MOESM1_ESM.pdf]

Spinning-disc confocal microscopy in the second near-infrared window (NIR-II)

Vitalijs Zubkovs[1], Alessandra Antonucci[1], Nils Schuergers[1], Benjamin Lambert[1], Andrea Latini[2], Raino Ceccarelli[2], Andrea Santinelli[2], Andrii Rogov[3], Daniel Ciepielewski[3], and Ardemis A. Boghossian[1]

[1]Institute of Chemical Sciences and Engineering (ISIC), École Polytechnique Fédérale de Lausanne (EPFL), Lausanne, CH-1015, Switzerland

[2]CrestOptics S.p.A., Rome, 00167, Italy

[3]Nikon GmbH, Swiss Branch, Egg, 8132, Switzerland

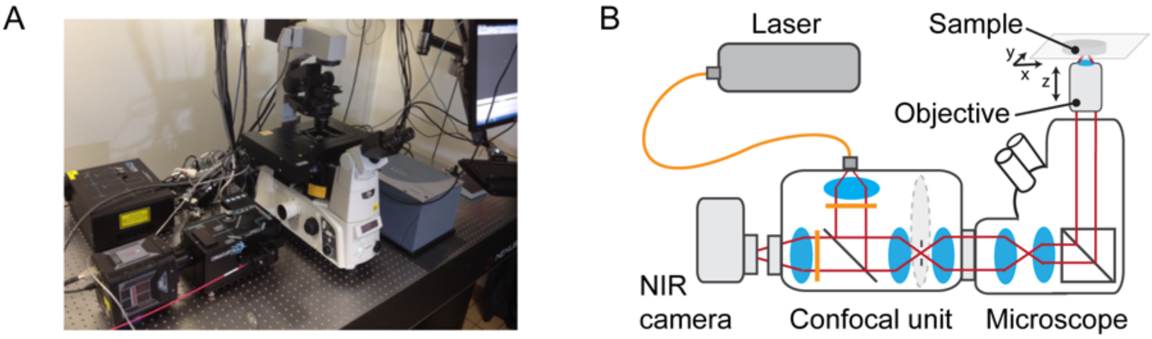

Figure S1. (a) An image and (b) optical layout of the confocal spinning-disc NIR microscope.

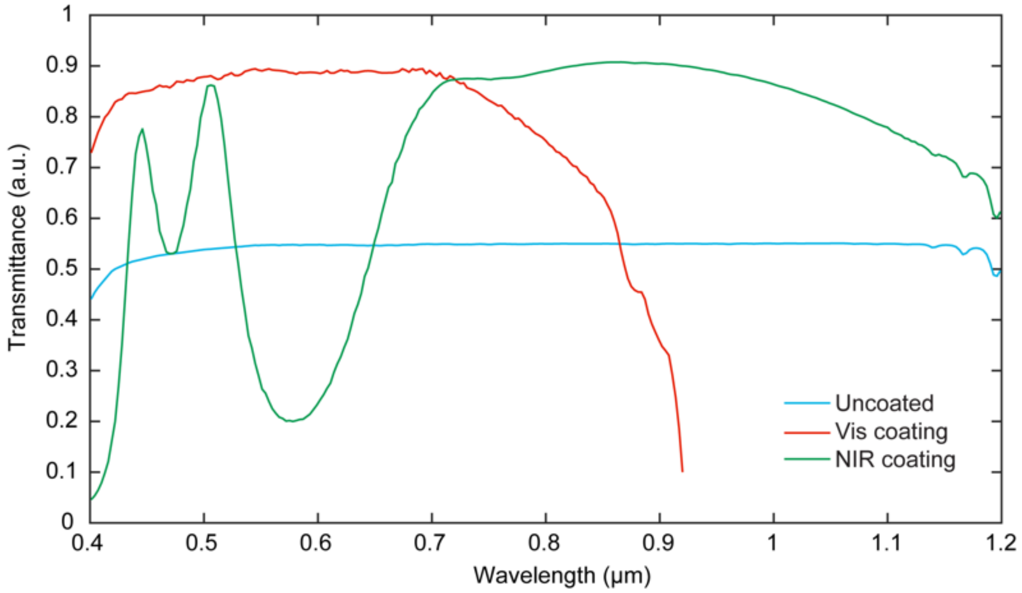

Figure S2. Transmission of the excitation light path for a standard production unit, IR optimized unit, and IR unit with uncoated lenses. Glass absorption, Fresnel losses, and mirror coatings are considered.

Table S1: Data obtained from fitting the lateral FWHM of the NIR beads using PSFj software in the wide-field and confocal microscope settings.

Nb. of beads	Wide-field FWHMx (µm)	Confocal FWHMx (µm)
1	0.823	0.377
2	0.542	0.758
3	0.524	0.456
4	0.523	0.497
5	0.591	0.337
6	0.471	0.556
7	0.526	0.415
8	0.525	0.466
9	0.449	0.451
10	0.450	0.411
11	0.438	0.353
12	0.492	0.401
13	0.445	0.377
14	0.448	0.406
15	0.434	0.507
16	0.506	0.389
17	0.874	0.387
18	1.095	0.402
19	0.619	0.384
20	0.635	0.398
21	0.790	0.526
22	0.720	0.466
23	0.641	0.798
24	0.588	0.539
25	0.481	0.542
26	0.601	0.387
27	0.475	0.768
28	0.569	0.670
29	0.691	0.540
30	0.470	0.588
31	0.515	0.486
32	0.572	0.405
**Average FWHM (µm)**	**0.6**	**0.5**
**Standard deviation (µm)**	**0.1**	**0.1**

Table S2: Data obtained from fitting the axial FWHM of the NIR beads using PSFj software in the wide-field and confocal microscope settings.

Nb. of beads	Wide-field FWHMz (μm)	Confocal FWHMz (μm)
1	1.115	0.481
2	0.982	0.678
3	1.019	0.550
4	1.154	0.568
5	0.953	0.591
6	0.937	0.843
7	1.141	0.516
8	1.067	0.556
9	0.814	0.517
10	1.069	0.601
11	0.878	0.498
12	1.238	0.531
13	0.971	0.513
14	1.066	0.581
15	1.113	0.585
16	1.319	0.515
**Average FWHM (μm)**	**1.1**	**0.6**
**Standard deviation (μm)**	**0.1**	**0.1**

$$SNR = \frac{n_s - n_b}{\sigma_b} \quad (S1)$$

The signal-to-noise ratio (SNR) was calculated according to Equation S1[1], where n_s is the intensity of a single NIR bead, n_b is the mean intensity of the background, and σ_b is the standard deviation of background intensity.

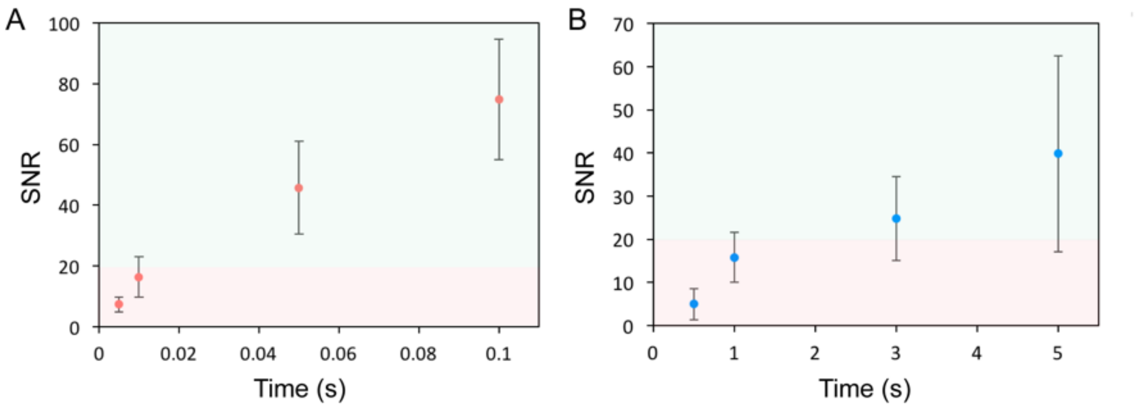

Figure S3: Signal-to-noise ratio (SNR) dependence on a frame question time for (a) single NIR beads and (b) single SWCNTs. Images were recorded in the confocal configuration (excitation at 780 nm, laser illumination power 1.8 W·cm^{-2}). Images that have SNRs greater than 20 (indicated in the green region) are considered suitable for fluorescence microscopy measurements.

Reference

1. Liu, Z. *et al.* Quasi-confocal, multichannel parallel scan hyperspectral fluorescence imaging method optimized for analysis of multicolor microarrays. *Anal. Chem.* **82,** 7752–7757 (2010).
